# Conserving a sustainable urban environment through energy security and project management practices

**DOI:** 10.1007/s11356-022-21721-w

**Published:** 2022-07-14

**Authors:** Rashid Maqbool, Emily Jowett

**Affiliations:** Department of Mechanical and Construction Engineering, Northumbria University, Newcastle upon Tyne, NE1 8ST UK

**Keywords:** Energy security, Climate change, Urbanisation, Project management practices, Sustainable construction

## Abstract

Construction has been highlighted as one sector that could reduce its effect on the environment by implementing more sustainable methodologies. However, there are many different challenges preventing sustainable construction practice, and despite existing research providing advice for carbon emission reduction, these practices are being somewhat neglected. The purpose of this research is to investigate the challenges surrounding sustainable construction practice and the current knowledge on energy security and project management practices that can aid climate change mitigation. The design methodology of this study involves a thorough literature review of existing knowledge on the subject matter followed by primary research. The survey conducted collected quantitative data of 160 valid responses via an online closed ended questionnaire using snowball sampling methods from the construction and engineering professionals working in the UK construction industry. The study found that most participants agree with the encouragement of sustainable construction practices, but are generally unsure about what they can do. Factors such as government involvement and organisation culture have some significance but will rely on further research in order to assess specific influencing variables. The study contributes to existing research on factors surrounding project management and identifies and plethora of areas of improvement, that can be formed into a holistic approach to the current construction industry practice.

## Introduction

Globally, urban areas are home to over half of the human population, generating an estimated 75% of the whole planet’s energy use and Green House Gas (GHG) emissions combined (Gouldson et al. 2016). Rapid population growth and urbanisation specifically within developing countries has led to concerns about high-carbon emissions and the use of non-efficient fuels (Maqbool and Sudong 2018; Gouldson et al. 2015). Energy, transport and construction processes are still largely contributing to high carbon emissions, inefficient fuel processes and waste (Ji et al. 2018; Mao et al. 2013). Based on this knowledge, cities with densely populated communities must act responsibly towards the reduction of emissions and the adoption of high-carbon fuel alternatives. Concerns are rising about climate change and energy security as numerous countries are choosing high-carbon options, focusing on the increase of economic growth over sustainability (Le and Nguyen 2019). Electrification via low-carbon and renewable energy alternatives could be the best strategy moving forwards; however, there are currently several barriers preventing action (Stewart et al. 2018).

Construction has been highlighted as one sector that could reduce its effect on the environment by implementing more sustainable methodologies (Maqbool et al. 2022a, b). However, there are many different challenges preventing sustainable construction practice, and despite existing research providing advice for carbon emission reduction, these practices are being somewhat neglected (Darko et al. 2019). State-level enforcement and government policies are said to be a vital force in the shift towards sustainability, lack of financial incentives and current investments in high-carbon energy supply are also contributing to the problem significantly (Esmaeili et al. 2020).

Though there is much more research available to be considered (Ahmad et al. 2019; Hossain et al. 2020; Liu et al. 2015), from this review, it is clear that there are several major challenges in reducing carbon emissions and working in more sustainable ways. Looking forward, research needs to focus on the transition from high-carbon fuels to electrification so that rapidly urbanising areas may develop in a greener way. Since the current construction sector have much room for improvement, a culture of more sustainable practice needs to be cultivated.

Project management practice in the construction sector is said to be a strong influencer on the overall sustainability of its projects (Liu et al. 2020). Project management is generally goal-oriented and relies on the dynamic integration and partnership of multiple organisations during a single project. In a project management culture that values reflection, practices will continue to improve for the better (Kozak-Holland and Procter 2014) and more increasingly, project managers are using integrated software such as Building Information Modeling (BIM) to increase the overall sustainability of a project (Olawumi and Chan 2018). Since many project stakeholders are often involved in construction projects due to their ever-increasing complexity, it has been suggested that project managers can therefore influence the practice of more sustainable methods of construction practice (Liu et al. 2020). By exploring into energy security and sustainable urban development, specifically focusing on project management practice, we can reaffirm previous knowledge and assess how accurate some current theories are, providing a holistic awareness and potential ideas for further research.

The aim of this study is to outline key elements within sustainable urbanisation to develop existing knowledge on the subject and provide a more holistic understanding of how to encourage sustainable construction practice. The study will discuss the role of the project management practices within the construction sector, focusing on how it can increase sustainable practice. In summary, the research question is therefore:

Considering the responsibility of the construction sector in the context of climate change mitigation and energy security for sustainable urbanisation, how can primary research expand the knowledge on sustainable construction project management culture?

This study will cover the significance of contributing factors within sustainable construction development. It will survey individuals with knowledge of the construction industry in order to develop existing research. The objectives of this study are the following:

- Identify and discuss environmental challenges surrounding sustainable urbanisation, and the role of construction practices in climate change mitigation and ensuring energy security.
- Understand the significance of the role of project management practices in the construction industry, and discuss key factors in project management that may influence more sustainable urban development.

This research will provide a base for the practicing engineering managers within and outside the construction industry to make sustainable practices to deal with the environmental challenges surrounding urbanisation for climate change mitigation and energy security to the wider community. More specifically, the study would support the managers and project leaders to choose wisely whilst deciding on the nature of project and then choice of the project practices which could not affect negatively for the sustainable urbanisation. This may be done through getting to know with the sustainable resilience, knowledge of sustainable project management practices, training on the modern methods of construction and finally through ensuring future oriented decision for sustainable communities (Maqbool and Amaechi 2022).

**Fig. 1:**
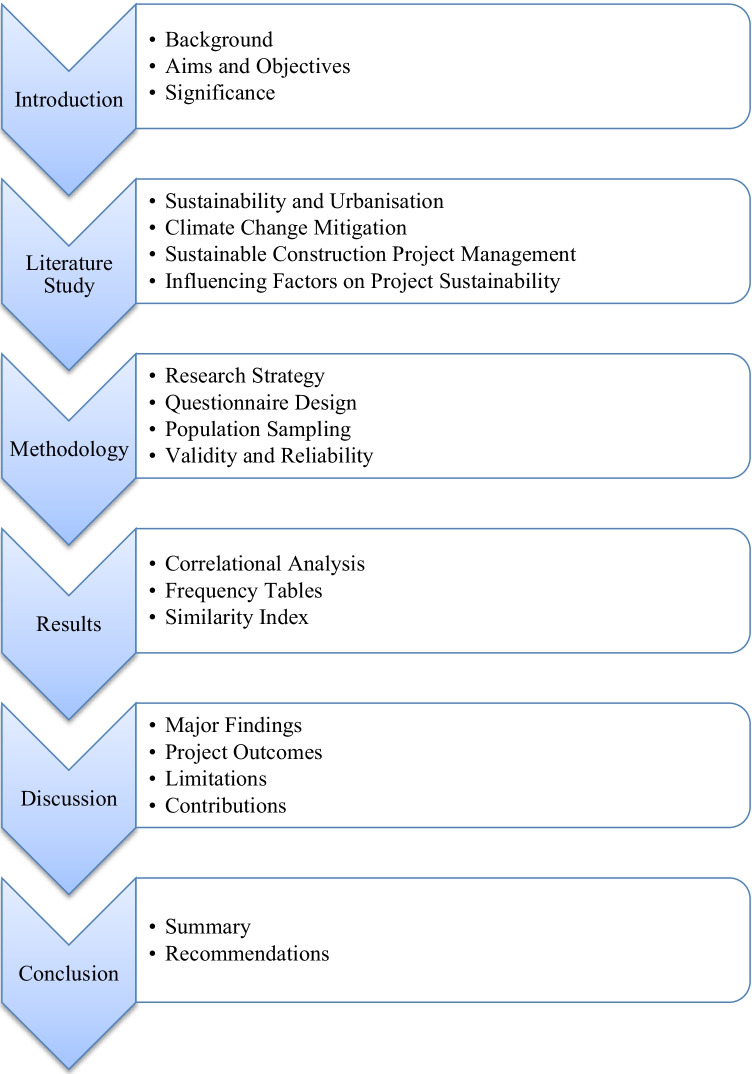
Structure of the study

This study will continue by thoroughly analysing current literature on the topics of sustainable urbanisation, challenges surrounding the topic, project management practice and potential influences on sustainable construction culture. The methodology section will discuss the process of the investigation including the development of the questionnaire, which draws from the literature reviewed. The results of the questionnaire will be then be analysed and interpreted in the discussion. Figure 1 outlines the format of this study report.

## Literature study

This literature study covers the topics of Sustainability and Urbanisation, Climate change mitigation and sustainable construction project management and explores studies on factors that influence project management sustainability factors.

### Environmental issues linked to urbanisation

The top ten most polluted cities on earth include six of India’s cities (IQAir 2021). Of the largest growing countries in Asia, the urbanisation of India in particular is the most environmentally alarming; with the second highest number of massive cities, almost 35% of India’s population now live in urbanised areas (O'Neill 2022). The environmental impact of this means that India is facing pressure to tackle its use of resources such as materials and energy, as well as reducing harmful emissions into the air and water supplies. Through the primary focus on economic growth and rapid urbanisation, it seems that the importance of sustainability has been somewhat disregarded, and the impact of such dramatic growth now means that there is an urgency to change the efficiency of resource utilisation, urban planning, environmental conservation policies, Green House Gas (GHG) emissions, and general cultural attitudes (Liu et al. 2014). Previously, out of ten most polluted largest cities seven were from China (Liu et al. 2014). Furthermore, almost five hundred of China’s largest cities failed to meet international air quality standards recognised by the World Health Organisation (WHO) (Liu et al. 2014; Zhang and Crooks 2012). However, China worked very hard for its urbanised sustainability, and the result is that at the moment there are only two Chinese cities among the top fifty most polluted cities in the world (IQAir 2021). So, it means that the environmental issues are linked with the urbanised pollution, and its sustainability can be ensured by focusing on the control on rapid urban population, urban planning with the urgency to change the efficiency of resource utilisation, environmental conservation policies, Green House Gas (GHG) emissions, and general cultural attitudes.

### Sustainability and urbanisation

Countries that are more economically developed, such as the USA, have put more emphasis on sustainability as the country evolves; however, countries such as India that are still described as ‘developing’, albeit rapidly, are focusing more on economic development rather than overall sustainability and the effects on the environment (Arditi et al. 2017; Konanahalli and Oyedele 2016). Other studies argue that sustainability policies do not consider the economic impact enough and are not encouraging the generation of jobs (Stewart et al. 2018); despite the impact that construction has, infrastructure is widely known as the driving force of industrialization, and a vehicle for development of any country (Chukwuji et al. 2020). Generally speaking, areas that will struggle most against rapid urbanisation are those that are more vulnerable due to existing issues with unemployment and poverty, this includes cities in South Asia, Africa and the Southern Americas (Stewart et al. 2018). The concept of *Green Growth* is explored as a paradigm that aims to increase economic stimulation whilst simultaneously decreasing wasted resources and environmental harm. Though the pressures of global warming and population increase are an international responsibility, cities will always be the most concentrated areas of consumption and waste production (Maqbool and Wood 2022).

Urban Sustainability frameworks developed by UNEP necessitate an integrative bottom-up approach, that requires sensitivity during application in local areas (Stewart et al. 2018). They involve inter-disciplinary coordination among political and legal institutions as well as technological sectors for a multi-dimensional strategy with maximum impact (Peter and Swilling 2012). The sensitivity to local requirements was deemed vital for success of the urban sustainability approaches put forward, particularly in low-income areas, stressing the need for frameworks to be holistic and flexible in nature (Hussain et al. 2022).

### Energy security and sustainable urbanisation

In less affluent and developing countries that have high prevalence of poverty, it has been estimated that the majority of their populations are living on a maximum of two dollars per day (Guriro et al. 2019). Energy provision is found to be ‘a key enabler’ for the alleviation and prevention of poverty (UNDP 2005). More specifically, modern methods of energy supply and access to electricity play a pivotal part in the sustainable development of emerging countries (Hussain et al. 2022; Maqbool et al. 2020a). The concept of *energisation* is a broader approach to electrification that focuses on improving energy provision and empowering communities in a way that encourages sustainable electrification and aims to reduce the reliance on high-carbon fuels (Nissing and von Blottnitz 2010). Research suggests that in some developing countries, carbon emissions are higher due to the proportion of high-carbon fuels being used compared to cost effective lower-carbon alternatives (Gouldson et al. 2016). Since we now rely so highly on power supply, it has been suggested that the low-carbon electrification of cities would be the most effective method to combat the environmental impacts of rapid urbanisation (Stewart et al. 2018).

In 2008, Alnaser, Flanagan and Alnaser reported that the impact of the global building sector consisted annually of massive environmental impacts such as energy use (42%), atmospheric emissions (40%), raw materials use (30%); lower yet still concerning forms of waste include use of land (12%), solid waste (25%), water use (25%) and water effluents (20%) (Alnaser et al. 2008). However, Chwieduk (2003) discussed how buildings could be made more energy-sustainable, through three main principles including energy-efficiency measures, sustainable design solutions, and renewable energy technologies. Whilst research shows that buildings can be developed to reduce waste and be more energy-efficient, years later, the global construction industry still needs to make many changes in practice for accumulative overall benefit and reduced environmental impact (Maqbool et al. 2022a).

### Climate change mitigation and sustainable urbanisation

Transport and construction have been identified as two major areas where carbon emissions can be reduced significantly in parts of Asia; however, in order for such countries to take action, their local governments need to be fully committed (Ji et al. 2018; Mao et al. 2013). Traffic management and infrastructure need to be replanned and better monitored, with the introduction of more affordable, fuel-efficient vehicles. Despite current knowledge, sectors are still not prioritising the reduction of waste and carbon emissions, and research is finding that low carbon alternatives are readily available but are simply not being invested in (Gouldson et al. 2016). It seems that action against climate change requires intensive coordination between many organisations within multiple sectors of industry. Interventions are needed from national state and local governments on policies regarding energy use in commercial industry, finance, residential housing sectors as well as land planning and overall economic development (Esmaeili et al. 2020). Some developing countries are still struggling to provide a secure energy supply to all areas; moving forward, the focus needs to be on providing low carbon energy supply for all, but most significantly at urban levels where populations are highly concentrated and rapidly growing (Maqbool et al. 2018; Hussain et al. 2022).

Metabolic models link the activities to the wastes, which can show professionals what to prioritise as the models consider the behaviour of the cities’ inhabitants and allows the setting of goals and the tracking of environmental indicators (Perrotti and Stremke 2020). Whilst waste management is a key part of sustainable living, a vital part of sustainable action needs to be concentrated on improving efficiencies in systems before the waste is created (Liu et al. 2014).

### Sustainable construction project management

#### Sustainable infrastructure

As identified earlier, transport and construction are the two main industries that have the highest potential for carbon emissions reduction (Liu et al. 2019). Furthermore, the need for sustainable infrastructure within the field of construction has widely been ranked as the most impactful on urbanisation (World Economic Forum (WEF) 2016). This is due to the great number of stakeholders involved in such projects, as well as the vast amounts of resources used during the completion of each one (Liu et al. 2020). Infrastructure often has a long lifespan, between fifty and a hundred years, which is more beneficial to the environment and inevitably those who will use it (Lee and Ellingwood 2017). Sustainable infrastructure therefore needs to be prioritised in urbanising areas, allowing the collaboration of different sectors to deliver such large projects through more sustainable methods. The more stakeholders that engage in more sustainable practices, the more likely we are to see a culture of change within the construction sector and other closely connected industries.

#### Project management practices

The practice of Project Management has been described by the PMI (Project Management Institute 2013) as the application of knowledge, skills, tools and techniques onto project related activities in a goal-oriented way in order to establish a project success. A study by Hwang and Tan (2012) outlined the most critical success factors linked to project success. They highlighted the top five most critical factors for project success:

1. Decision-making effectiveness (project-management-related)
2. Project’s adequate funds/resources (project-related)
3. Top management support (project-management-related)
4. Availability of experienced managers and skilful workforce (contractor-related)
5. Coordination between all participants (project-manager-related).

Fong and Lung (2007) identified a positive relationship between interorganizational teamwork and project success in the construction industry. They measured this relationship through assessment of inter-contextualized cultural factors and task performances.

Project Management Sustainability can be described as project delivery that is supported by planning, monitoring and control processes that take environmental, economic and social factors into consideration throughout the lifecycle of the whole project (Sabini et al. 2019). Since project management is so closely linked to project success, research suggests that the strategy for constructing in more sustainable ways is through the Project Management Practices (PMPs) (Banihashemi et al. 2017).

### Influencing factors on project sustainability

Certain factors that influence sustainable construction practices have been identified through various research, including top managers’ leadership (Meng et al. 2015), stakeholder engagement (Bal et al. 2013), project management knowledge and skills (Hwang and Ng 2013) and greening PMPs for sustainable construction (Robichaud and Anantatmula 2011). This research will seek to explore which are the key influencing factors.

#### Organisational culture and project sustainability

Organisational culture has been outlined as the most significant feature to distinguish excellent companies from the rest (Cameron and Quinn 2011), since it influences the whole operation of a company through its atmosphere. A culture can influence the use of non-renewable resources and the utilisation of low-carbon fuels (Liu et al. 2020). Gimenez et al. (2012) suggests that environment-friendly programmes implemented through organisational culture constraints have positive impacts on project sustainability. Cultural development should therefore take priority over other corporate strategies (Arditi et al. 2017). O'Brien (1999) reported many companies incorporating environmental management into the core of their business strategy. Liu et al. (2020) states that well-established contemporary construction companies should incorporate sustainability into their culture, which would in turn, influence the regular behaviours of other organisations and their affiliates. Culture within organisations is something that is formed by its leadership values, routines and prioritised functions (Liu et al. 2020). In construction, the outcome of any project is dependent on the culture of the organisations involved; it translates the vision of the project and therefore has been outlined as a key influencing factor (Liu et al. 2020). Another study by Schein (2010) also found that the performance of organisations is affected by the intensity of its culture. Naoum et al. (2015) elaborates on this within the field of project management stating that the decision-making process is affected by the goals of the organisations involved, thus creating a specific project management culture.

As previously discussed, establishing a fully integrated and comprehensive approach that is flexible for global organisations, as opposed to a rigid standard of rules, can guide industries towards change (Liu et al. 2014). Since it is much more difficult to measure the sustainability of infrastructure projects delivery due to their complex nature (Liu et al. 2014), sustainable methodologies need to be integrated into all areas of the industry first through collective cultural practices. When discussed through lean construction practices, the strategy of reflection was adopted as a process of learning, knowledge sharing and continuous improvement (Kozak-Holland and Procter 2014). Through learning across various projects and sharing that experience collaboratively, project management and construction practices may have the potential to develop more mindful and efficient ways of working.

#### Project management success factors and project sustainability

A study by Ogunde et al. (2017) explored the challenges facing construction project management systems in Nigeria. They identified that if a construction project manager is to successfully implement project management practices, it is necessary to understand and possess management skills in planning, organizing, commanding, coordinating and controlling (Ogunde et al. 2017), which could suggest that further training programmes might be beneficial, or that current academic programmes should be better enriched.

The study categorised the main challenges witnessed during construction projects into six related to Project Management, Client, Consultant, Supplier, Construction team and External factors. The main challenges identified were the following:

- Lack of client involvement in making decisions.
- Provision of substandard materials.
- Design error.
- Lack of effective communication.
- Poor treatment of workforce.

After going through the literature review, a list of project management success factors has been identified which could be beneficial for the sustainable urbanisation. The list of project management success factors with relevant sources of references is presented in Table 1.

**Table 1:** Project management success factors

Critical success factors	Source (s)
Support from senior management	(White and Fortune 2002); (Ofori 2013); (Jha and Iyer 2006); (Alias et al. 2014);
Commitment of all project participants	(Chua et al. 1999); (Maqbool et al 2022b); (Chan et al. 2001); (Munns and Bjeirmi 1996)
Adequate communication channels	(Maqbool et al 2018); (White and Fortune 2002); (Ofori 2013); (Chan et al. 2001)
Effective control, such as monitoring and updating plans	(Chan et al. 2001); (Chua et al. 1999); (Ofori 2013); (White and Fortune 2002); (Maqbool and Amaechi 2022)
Adequate financial budget	(Chan et al. 2001); (Ofori 2013); (White and Fortune 2002); (Maqbool et al 2020b)
Skilled designers	(Chua et al. 1999); (Maqbool and Amaechi 2022)
Skilled project managers	(Chan et al. 2001); (Ofori 2013); (Jha and Iyer 2006)
Troubleshooting	(Belout and Gauvreau 2004); (Ofori 2013)
Project team motivation	(Chua et al. 1999); (Ofori 2013); (Maqbool et al 2022b)
Strong/detailed plan effort in design and construction	(Chan et al. 2001); (Munns and Bjeirmi 1996)
Effective feedback	(Chan et al. 2001); (Ofori 2013); (White and Fortune 2002); (Maqbool et al 2018)

#### Stakeholders and integrative technologies and project sustainability

The fragmented nature of the construction industry is made up of project stakeholders (Maqbool et al. 2022b). Each project interacts with many key parties or organisations. Eberendu et al. (2019) reported that successful project operation is dependent on stakeholder involvement. Furthermore, Bal et al. (2013) reported on the effect of stakeholders on sustainable construction strategies. The study suggests that when the key members involved in a project have a harmonious relationship, the project as a whole is more likely to succeed due to the experience and shared knowledge that is gained. So, if managed efficiently, stakeholder collaboration could be seen as a tool to better achieve project sustainability satisfaction.

Harris et al. (2021) discuss the issues with stakeholder engagement and suggest that poor communication between the necessary project members reduces meeting satisfaction and overall objective comprehension. With less information, stakeholders are forced to make assumptions or decisions, and that in turn causes mistakes throughout the project lifecycle.

A study by Persson (2009) revealed that the key stakeholders involved in a selection of construction projects were not well-enough informed about sustainable construction practices such as energy-efficiency measures (less than 31%), sustainable and renewable design solutions (less than 13%). Since stakeholder participation is so vital, it is not surprising that their knowledge base and efficiency have an overall effect on its sustainability in practice. Persson (2009) reported that the knowledge on energy-efficiency is linked to job role and experience in the Saudi building industry. They found that education level and experience in the industry had a positive relationship on interest in sustainable building. The study highlighted that clients and contractors were less likely to have sufficient awareness and recommends that stakeholders take part in relevant training programmes (Alrashed and Asif 2014).

Integrated software such as Building Information Modeling (BIM) platforms have been heralded for its engagement and collaboration of stakeholders. Olawumi and Chan (2018) surveyed international construction experts commended BIM adoption and implementation for its assistance with sustainable practice management and overall project success. They discuss that important knowledge on sustainability, even when obtained, is not being utilised effectively until tasks are implemented. Since a platform such as BIM has the ability to organise and manage stakeholders through multiple layers of operation, operations can run more efficiently, reducing mistakes and reducing waste.

#### Policy factors preventing sustainable practice

In the analysis of present literature, several limiting factors have been identified as preventing sustainable practices. Barriers include a lack of shared values between city governing policies, limited local focused adaptations, poor implementation and maintenance of sustainable practices as well as poor initial planning of cities (Maqbool and Amaechi 2022). Sustainability seems to come second place to economic growth and is not being considered during the development of smaller cities into larger ones (Liu et al. 2014). Government involvement appears to be the common factor in the lack of low carbon alternative consideration when it comes to energy supply (IPCC 2015). Furthermore, it is vital that governing bodies act and recognise this importance so that policies can be implemented urgently. In some cases, this is made more difficult because powers are found to be under-developed, absent, diffused or fragmented (Gouldson et al. 2016). Therefore, the governance arrangements needed in order to explore and implement lower carbon electricity solutions are lacking or ineffective. Support and awareness need to be provided in order to secure these changes in multiple sectors and decarbonise the electricity sector. Though it is suggested that national climate commitments are readily achievable, they ‘lack ambition’ and governments “choose only to exploit those options that are economically attractive” (Gouldson et al. 2016, pp 16). What needs to be provided is a financial incentive for authorities to cooperate with low carbon initiatives, as well as mutual collaboration with international organisations. In order for climate change mitigation to progress, attractive low-carbon measures such as waste reduction and utilisation require significant financial investment (Maqbool et al. 2020b). Many cities still rely on high carbon options; especially in those that are growing rapidly, the need for change is urgent, and is highly dependent on political involvement at an international level (Reckien et al. 2018).

## Methodology

**Fig. 2:**
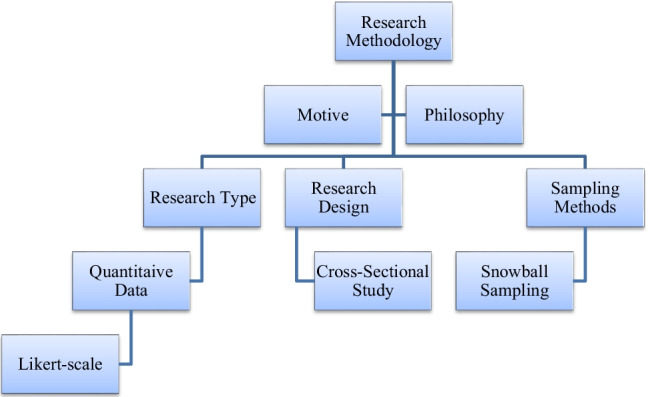
Research methodology breakdown diagram

The methodology section covers the scientific approach behind the study, moreover the research strategy that was used including the motive and philosophy for the research as well as information on how the study was designed, structured and taken place. Population sampling methods and participant recruitment are also covered in this section. Figure 2 highlights a top to bottom complete details of the research methodology involved in this research.

### Research motive

The motive behind this questionnaire was drawn from the examination of the literature present in the review (Gouldson et al. 2016; Gunduz and Almuajebh 2020; Ofori 2013). There are studies that suggest more specific actions that companies can take to work in more sustainable ways (Maqbool et al. 2018; Maqbool and Amaechi 2022). However, they feel somewhat juvenile when compared against the vast challenges that the world still faces when it comes to climate change mitigation and global energy security. Since so much research already exists, it is necessary to collate information in a holistic way to ask questions about the kind of solutions that may be beneficial moving forward. If project management has some influence on how construction can make strategic changes for the better, then research must be directed in order to bring about cultural change in practice.

### Research philosophy

Research philosophies, as discussed by Saunders et al. (2009), are the beliefs about how a theory should be studied, it determines the way in which a researcher would explore, gather and analyse data. The Positivist philosophy approaches theories through the lens of the natural sciences (Bryman 2015). Positivism aids the investigation of research questions in a purely objective way (Mackey and Bryfonski 2018), which relates to the research type because the questionnaire was designed to gather high-quality numerical data.

### Research type

There are two types of research collection, named qualitative and quantitative. The primary research conducted in this study gathered quantitative data. This is because the questionnaire was developed around the collection of numerical data using the five-pointer Likert scale ranging from strongly disagree to strongly agree valued between one and five. The use of the word quantitative emphasizes quantification and statistical analysis of the high-quality data collected (Bryman 2015).

## Research design

Following a thorough examination of the literature, a closed, structured questionnaire was developed to collect numerical, quantitative data. The survey included a total of 160 samples within the period of research. This makes the research design a cross-sectional study because more than one case at one single point in time was collected (Bryman 2015).

The sample included industry professionals as well as researchers with knowledge of the design and construction industry. It was necessary for the study to use the cross-sectional design because time and budget was limited; however, this means that the results are all current; alongside the quantification of data through the five-pointer Likert scale, it ensures reliable analysis and allows the opportunity for thorough discussion following this (Creswell and Hirose 2019).

### Questionnaire design

The questionnaire was sectioned into different categories, the first being some demographic based questions; however, all data was kept anonymous. The other categories covered the topics of Energy, Organisational factors, Project Management factors, Stakeholder factors, Policies and Sustainable Urbanisation. No sensitive topics were addressed throughout. The questions were chosen to provide insight into what challenges might be preventing the uptake of renewable energy sources and sustainable practices, as well as potential solutions that could be available. The study aimed to assess what factors are significant in the influence of sustainable practices. The literature review revealed that there are many overarching influences and variables involved. Table 2 lists the key studies discussed in the literature review that the questionnaire design was developed from.

**Table 2:** Questionnaire design references

Questionnaire design table			
Category	Number of questions	Themes	Source (s)
Demographics	4	Gender Level of education influence Designation Experience	
Energy	9	Use of low-carbon alternatives compared to high-carbon fuels Electrification vs energisation	(Gouldson et al. 2016) (Nissing and von Blottnitz 2010) (Stewart et al. 2018)
Organisational related Factors	9	Awareness of sustainability Top management leadership Support from senior management Organisation culture Project team motivation	(Alrashed and Asif 2014) (Meng et al. 2015) (Gunduz and Almuajebh 2020) (White and Fortune 2002) (Jha and Iyer 2006) (Liu et al. 2020) (Chua et al. 1999)
Stakeholder related Factors	9	Stakeholder engagement Stakeholder knowledge/ experience of energy efficiency and sustainability Adequate communication channels Client Involvement Coordination between all participants Effective feedback	(Bal et al. 2013) (Ogunde et al. 2017) (Alrashed and Asif 2014) (Persson 2009) (White and Fortune 2002) (Chan et al. 2001) (Ogunde et al. 2017) (Gunduz and Almuajebh 2020) (Chan et al. 2001) (White and Fortune 2002)
Project Management related Factors	9	Effective control, such as monitoring and updating plans Project management knowledge and skills Availability of experienced managers and skilful workforce Project’s adequate funds/resources Decision-making effectiveness Passive participation from the PM Project management influence on sustainability	(Chan et al. 2001) (Chua et al. 1999) (White and Fortune 2002) (Hwang and Ng 2013) (Chan et al. 2001) (Jha and Iyer 2006) (Gunduz and Almuajebh 2020) (Gunduz and Almuajebh 2020) (Chan et al. 2001) (White and Fortune 2002) (Gunduz and Almuajebh 2020) (Ogunde et al. 2017) (Liu et al. 2020)
Policy related Factors	9	Increased sustainability content on Higher Education courses Encouragement of sustainable construction practices Incorporation of Lean principles into practice BIM adoption and implementation for sustainable practice Insufficient government action	(Alrashed and Asif 2014) (Robichaud and Anantatmula 2011) (Kozak-Holland and Procter 2014) (Olawumi and Chan 2018) (Gouldson et al. 2016)
Sustainable Urbanisation	9	Sustainable infrastructure Rapid urbanisation Economic development and prioritisation of sustainability Urban responsibility	(Chukwuji et al. 2020) (Lee and Ellingwood 2017) (Liu et al. 2014) (Stewart et al. 2018) (Arditi et al. 2017) (Konanahalli and Oyedele 2016)

### Population sampling

#### Participant recruitment

This study uses the Snowball sampling method. These methods allow the study to approach participants that represent a certain population. This means that the sample will be representative of a specific trait to suit the needs of the study (Acharya et al. 2013). The Snowball Sampling method works effectively in studies where an individual participant is not assumed to represent the whole speech community. This method then relies on participants’ networks and connections in which they are asked to share the study with affiliated participants in order to gather a larger sample to partake (Bryman 2015).

Subjects were approached and recruited via the researchers from different Universities currently studying or who have previously studied on the MSc Construction project management courses or the aligned courses alumni. The questionnaire was sent out electronically via email to researchers of universities, as well as professionals currently based in the design and construction sector. Snowball sampling was generally used following this, as those surveyed were able to share the questionnaire with other industry professionals. Any organisations affiliated with those researchers that have project management experience may have also been approached. At the start of the questionnaire, a brief paragraph explained the nature of the study and the concept of anonymity so that participants were informed before participation.

#### Sampling stratification and size

The final sample size was 160 participants, which was stratified according to job role labelled in the demographics Table 3 as ‘Designation or Equivalent’, current level of education, experience in construction industry (years) and gender. The sample is intended to represent variation across the design and construction industry, including minorities to enhance the validity of the results. The demographics frequencies are presented in Table 3. The values highlighted in green are the highest frequencies and those in red are the minorities for that category. The highest responses for demographics were Males, Project Managers, educated at Masters level, with 5–10 years in construction industry. Whilst the sample was collected from the UK, other countries may have been reached as the questionnaire was sent over various virtual platforms, e.g. email and social media such as LinkedIn due to the Covid-19 pandemic. However, it was made sure that all the respondents have or had a knowledge and experience of UK construction industry, as some of the respondents might have worked in the UK construction industry previously and employed in overseas industry now.

**Table 3:**
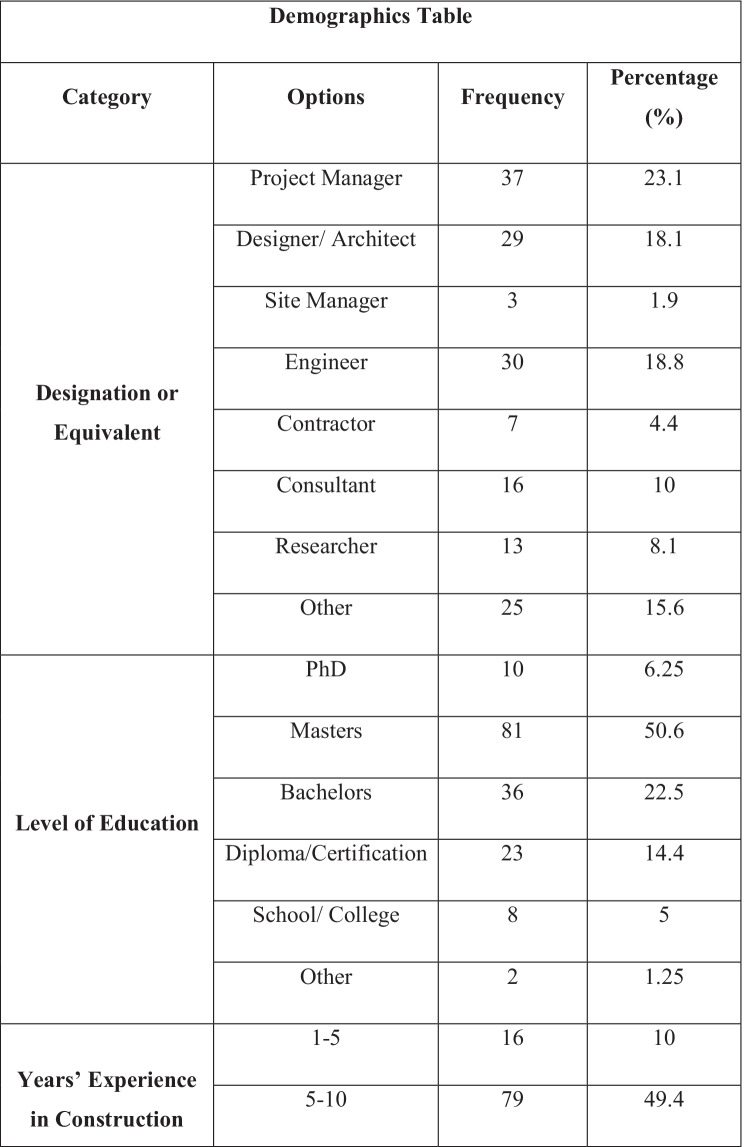
Demographic frequencies

### Validity and reliability tests

Validity and reliability statistical tests are utilised in order test the integrity of the conclusions that are generated from a piece of research and to make sure the findings are applicable to real world scenarios.

The questionnaire data from the survey website Jotform was exported as an Excel file. The Excel file once ready was imported into SPSS statistical analysis software so the data could be coded and analysed. No other software was necessary for the study.

#### Cronbach’s alpha

As shown in Table 4, a Cronbach’s alpha reliability test was carried out across the survey questions to assess the level of variance between the respondent answers. This also tested the internal reliability of the results. All six of the categories in the Table 4 show values which are > 0.7. According to Amirrudin et al. (2021), the acceptable Cronbach’s alpha value range from 0.6 to 0.7, whereas any value over 0.7 is considered as good Cronbach’s alpha value. Since the Cronbach’s alpha values are all above 0.7 which is close to 1, we can infer that the level of variance throughout the study is high and therefore the internal reliability is high. The highest value was 0.864 for stakeholder related values and has been highlighted in green.

**Table 4:**
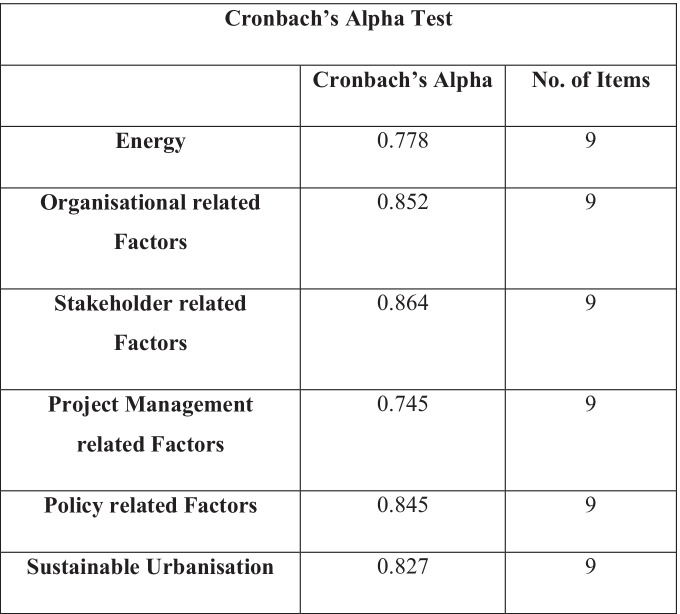
Cronbach’s alpha table

#### KMO and Bartlett’s test

A KMO and Bartlett’s test was completed through SPSS in order to detect the suitability of the data for structure detection.

If the KMO value > 0.6, this indicates that the test is useful (Glen 2016). Table 5 shows that the KMO measure number = 0.826, highlighted in green. This means that the data is well suited for factor analysis or principal component analysis, which will benefit the research and extract the most significant variables. If < 0.001, then we can reject the null hypothesis and accept hypothesis as value significant for Bartlett’s test of Sphericity (Tobias and Carlson 1969), because Table 5 shows significance value = 0.000, which is close to 0, and so there is some scope for dimensionality reduction, i.e. reducing the number of dimensions in data set.

**Table 5:**
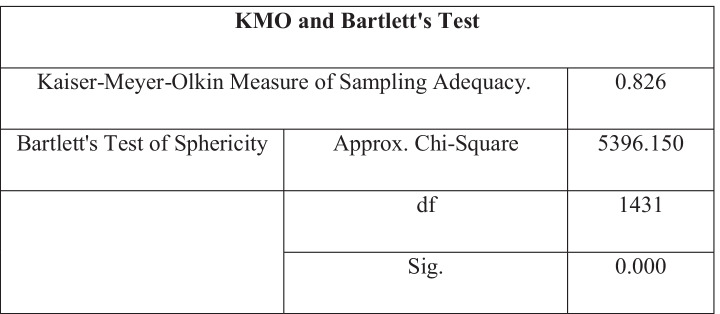
KMO and Bartlett’s test

### Factor analysis

Principal components analysis simplifies the input variables into components or factors. Factor loading is the correlation coefficient for the variable and factor and shows the variance explained by the variable on that factor. The factor loading component matrix was analysed for each of the 54 questions in the survey and extracted 13 potential components; however, not all values were significant. The factor loading of each variable above the acceptable minimum value of 0.5 was highlighted in Table 6 displayed. Table 6 shows that the highest values in the table were 0.674 for question E8, “The uptake of renewable energy sources over high-carbon fuels will lead to high energy security”, and 0.672 for question S7, “External stakeholders should have a say in the environmental effects of local projects”. Comparatively, the lowest value generated in the Table 6 is − 0.468 highlighted in blue for question P3, “Higher education should focus more on sustainable practice”. The values highlighted in red are those that are closest to 0, mainly organisational factors.

**Table 6:**
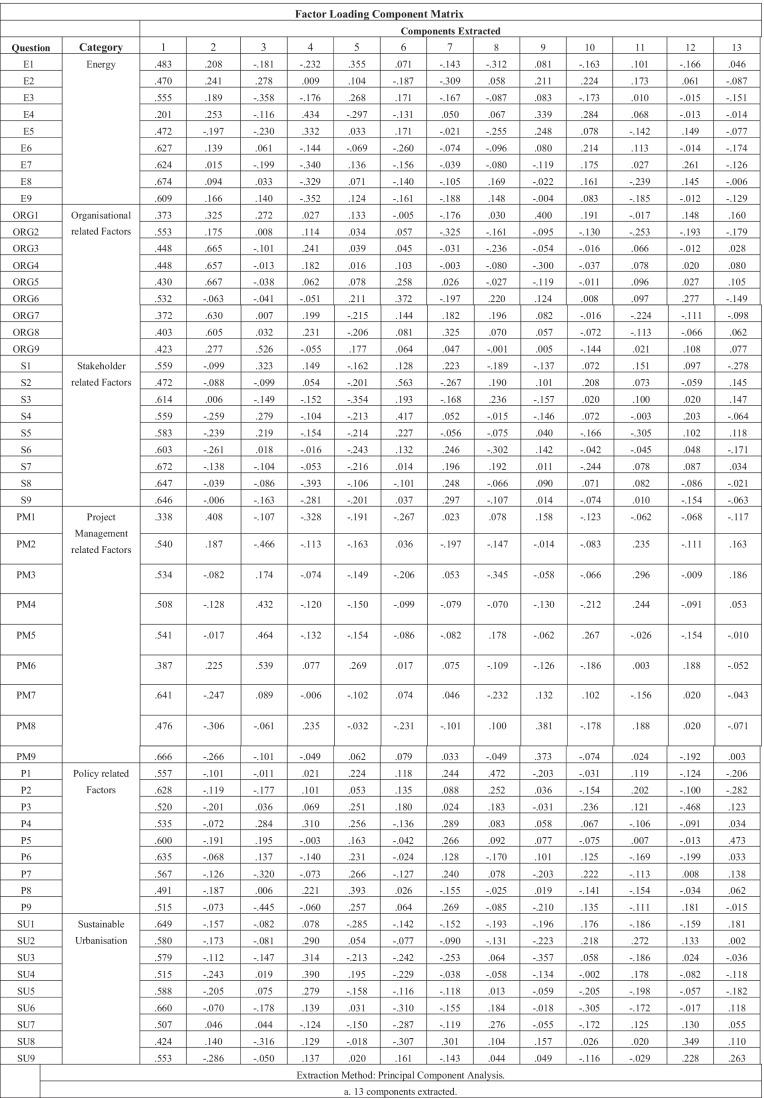
Principal components analysis

## Results analysis

### Correlational analysis

The results of the Pearson correlation test are listed in Table 7. The test assesses the significance of similarity in participant responses for each category against another. When the Pearson correlation value lies between 0.7 and 1, it means that the relationship between the variables is strong. If the *P*-value or significance value is > 0.05 (greater than 0.5), that means that the chance of error is more than 5%, and so the value is not significant. The results that are significant and show a high Pearson correlation value (over the minimum of 0.5) have been highlighted in green. This means that the most significant correlations were between Project Management factors and Energy factors, as well as Stakeholder factors and Project Management factors. Those values highlighted in red are identifying *P*-values that are close to 1, which means that the chance of error is higher and therefore the results for those sections are less consistent. The highest correlation values were for Stakeholder factors and Project Management factors with a value of 0.735 at significance level close to 0, i.e. < 0.001. The lowest Pearson correlation value was 0.001 for Experience in Construction Industry and Organisational factors, with a significance level of 0.995, which means there is a high chance of error.

**Table 7:**
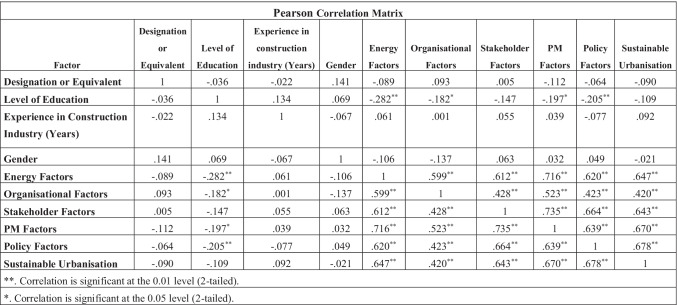
Correlation factor

### Descriptive statistics

Table 8 displays the mean answers and the standard deviation values for each question. Those highlighted in red are the lowest values, and those highlighted in green are the highest ones. Table 8 shows that the highest means were 4.56 for question E5, “I would use renewable energy sources more if they were cheaper or more readily available” and question ORG6, “Organisations have a responsibility to work more sustainably”. The lowest mean average values were 3.01 for question PM1, “There are enough skilled and experienced workers in construction” and 3.33 for question E4, “High-carbon fuels are more convenient than low-carbon alternatives”. The question with the lowest standard deviation was question SU9, “Urban planners and infrastructure developers should work to increase sustainability where possible”, with a value of 0.655. The highest deviation value is 1.276 for question SU8, “Reducing population sizes and/or birth rates is a potential strategy for climate change mitigation”, which means this question had the largest range in responses.

**Table 8:**
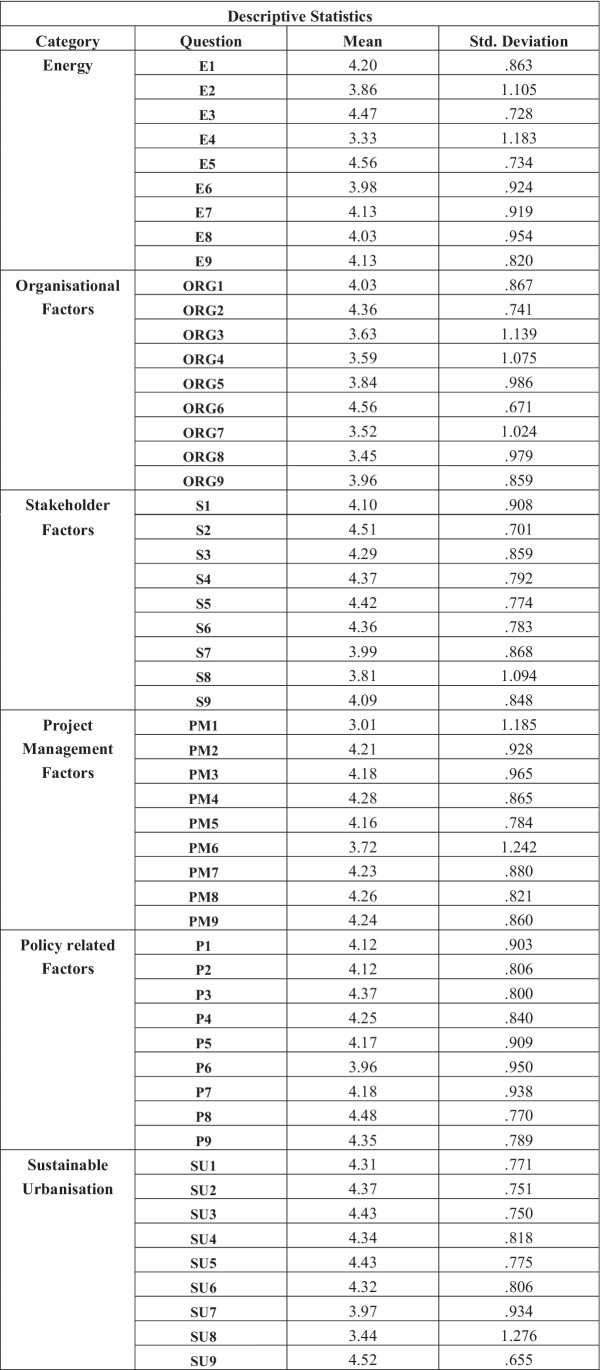
Frequency table

### Similarity index

Table 9 shows that the category with the highest standard deviation was Organisational factors as the mean value for this category was the lowest at 3.8896. Sustainable urbanisation had the highest mean value at 4.2362 and therefore also had the lowest standard deviation. The highest values are highlighted in green and the lowest are those highlighted in red.

**Table 9:**
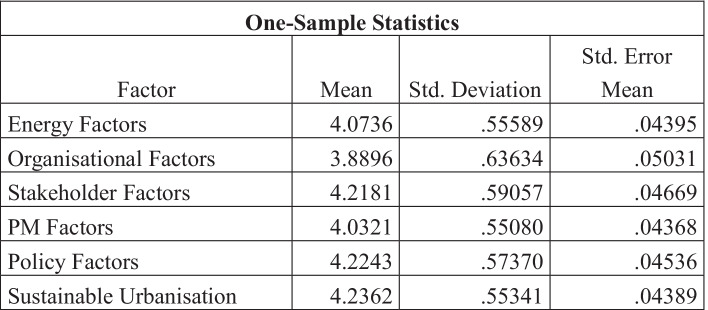
One-sample statistics table

Table 10 correlates with , and it also shows that the category with the highest similarity in responses is Sustainable Urbanisation and that with the lowest is Organisational Factors.

**Table 10:**
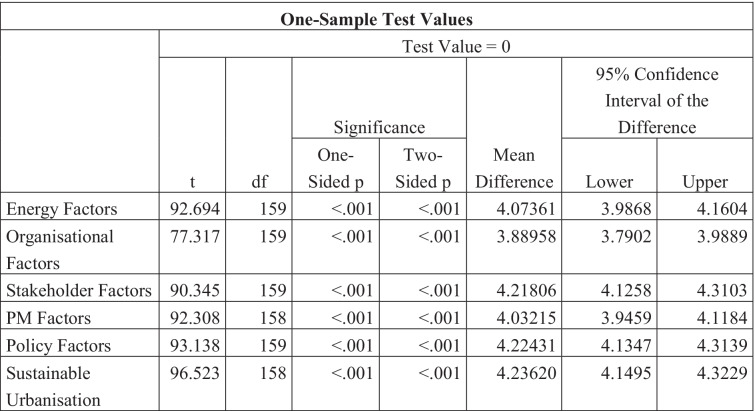
One-sample test values table

## Discussion of findings

It can be inferred that the questionnaire results gathered during the study have internal reliability because their Cronbach alpha values are all significantly close to the value of 1 (Amirrudin et al. 2021). Because the value is > 0.7, this means that the variance between the respondents answers is also high and therefore more reliable. The KMO test assessed the suitability for factor analysis and it was found to be appropriate. This is because the KMO value at 0.826 was > 0.6, which is close to 1 and therefore a strong indication (Tobias and Carlson 1969). Bartlett’s test value also proved to be significant which confirmed that data would be viable for correlational factor analysis. The factor analysis table displays the variance in answers depending on the variables applied and highlighted areas of significant correlation in the results. The factor loading of each variable above the acceptable minimum value of 0.5 (Shevlin and Miles 1998) was highlighted in . The table shows that the highest values in the table were 0.674 for question E8 (The uptake of renewable energy sources over high-carbon fuels will lead to high energy security) and 0.672 for question S7 (External stakeholders should have a say in the environmental effects of local projects). Comparatively, the lowest value generated in the table is -0.468 highlighted in blue for question P3 (Higher education should focus more on sustainable practice). The values highlighted in red are those that are closest to 0, mainly organisational factors.

A Pearson correlational analysis was then generated in order to assess which factors correlated most often at a significant level. The most significant correlational answers include Project Management factors and Energy factors, as well as Stakeholder factors and Project Management factors. The highest correlation values were for Stakeholder factors and Project Management factors with a value of 0.735 at significance level < 0.001. The lowest Pearson correlation value was 0.001 for Experience in Construction Industry and Organisational factors, with a significance level of 0.995, there is more inconsistency in the results. The demographics displays a majority of male candidates, at 68.8%, with 30% identifying as women. There is therefore a large disparity between the genders represented in the sample which could affect the real world application of the results. However, the UK gender diversity within the UK Construction industry in 2020 was 87.5% men and only 12.5% women (UKRI 2021); therefore, the sample is not too dissimilar to have external validity.

### Characteristics of sustainable urbanisation

The one-sample *T* test demonstrated that Sustainable Urbanisation had the most similar responses throughout the questionnaire, with a *T* value of 96.523, and therefore also had the lowest standard deviation at 0.55341. Sustainable urbanisation also had the highest mean value over the category at 4.2362. So, the findings of this study are well aligned with the Yigitcanlar and Teriman (2014), Stewart et al. (2018), who also highlighted the sustainable urbanisation as the key element to focus on. Question SU8, “Reducing population sizes and/or birth rates is a potential strategy for climate change mitigation”, had the lowest mean average value at 3.44 and the highest standard deviation of 1.276 for that category. This means that the variance in responses for that question was high and therefore the sample had conflicting opinions. This is a question that could be researched into further in depth; however, it is not directly linked to construction or sustainable management practice, as Gouldson et al. (2015) found the reduction in population size can impact on the climate change mitigation.

Question SU9, “Urban planners and infrastructure developers should work to increase sustainability where possible”, had one of the highest mean scores of 4.52 in the questionnaire, paired with one of the lowest deviations of 0.655. To summarise the category, the majority of the sample agreed with the statements involved in improving sustainability and approaches to urbanisation, and almost agree unanimously that urban planners and infrastructure developers should be working harder and increasing the efficient and sustainability of things such as infrastructure further. However, there is little light shed on whether these practices can actually be implemented (Maqbool and Sudong 2018; Hussain et al. 2022). Furthermore, due to the more complex nature of infrastructure projects compared to building projects, there needs to be extensive research and collaboration between disciplines in order to improve whole systems un the near future (Liu et al. 2020).

### Factors impacting sustainable urbanisation

#### Energy security impacting sustainable urbanisation

The correlational factor analysis showed that there is a high correlation between answers for questions related to energy, project management and stakeholder-related factors. We can infer from this that there is a strong positive correlation between perspectives on those sections and therefore those participants who’s responses correlated are more likely to have a greater understanding of energy related factors in construction project management. That being said, question E2, ‘I understand the concept of low-carbon electrification’, had a high standard deviation value and a mean average response of 3.86. This demonstrates that the general understanding of the concept of low-carbon electrification is averaging around neutral, suggesting that participants were not as informed as previously assumed. In order to get better energy solutions towards a sustainable environment, the public engagement and knowledge is utmost important (Liu et al. 2020; Maqbool and Amaechi 2022). Question E3, ‘I care about using renewable energy sources’, had a mean average response of 4.47 and a low deviation. So whilst participants are not fully aware of the concept of electrification through low-carbon methods, we can assume that they all have the capacity to learn because they care about the use of renewable sources. Moreover, due to unavailability of steady energy supply or high energy bills, people intend to think about alternative energy solutions (Maqbool et al. 2018).

#### Organisational factors impacting sustainable urbanisation

The results from the correlational factor analysis were somewhat surprising as they did not highlight any strong significant correlations with the organisational factors. Instead, there are several moderate correlations with organisational factors and the other variables involved; therefore, we can suggest that there is a loose relationship, which is significant however not very strong. It could mean that the participants have less experience in organisational cultures but are still educated on the subject areas. This assumption cannot be refuted by the demographics frequency table results. Those with 5–10 years of construction experience made up 79 of the 160 participants surveyed, at 49.4% that means almost half of the sample is relatively experienced to the industry. Alongside this, the sample was mostly made up of individuals with at least a Bachelor’s level degree or higher. A total of 22.5% of the sample had a Bachelor’s compared to 50.6% of the sample having a Master’s level degree. This means that we can assume that the participants have some knowledge on areas such as sustainability, but potentially less so when it comes to matters of work experience in the industrial practices towards sustainable urbanisation. It depicts that besides having some initial knowledge of sustainability, the experiences to apply technical and innovative solutions for sustainable urbanisation are of far more important (Bibri and Krogstie 2017).

Generally, the one-sample *T* test showed that the mean difference for organisational factors was slightly lower than the other factors, and the standard deviation was also slightly higher for that category that the others. This could mean that there was more variance in the answers given in that subject and therefore a more varied range in opinions. When individual questions were analysed, it was found that the mean responses were lower for questions ORG3, ORG4 and ORG5 respectively; “I feel supported by top management at work”, “Top management at my place of work cares about and encourages sustainable practice”, “My overall organisational culture values sustainability”. This explains why the average responses were lower for this category and not purely down to lack of participant experience. It is therefore recommended that organisations need to increase support provided to their staff and could indicate that organisational culture is an important factor. According to Roscoe et al. (2019), organisational culture and the support to staff for sustainability enhance their performance for sustainable development. The mean average answer for question ORG5 was 3.84, which demonstrates that not all participants perceived their organisations as ones that value sustainability highly. However, question ORG6’s mean average response of 4.56 shows us that the majority of the sample agrees that organisations have a responsibility to work more sustainably. Comparing these results, it can be suggested that the participants are aware of their organisational responsibility towards sustainable practice, but the organisations themselves and their top management need to take up more of that responsibility and actively takes steps to implement it.

#### Stakeholder factors impacting sustainable urbanisation

The frequency table shows that the questions in the Stakeholder category scored fairly highly with mean average values over 4. That means that the majority of the sample agrees with question S1 that “the sustainability of projects relies on the skills and knowledge of its stakeholders”. This supports studies by Alrashed and Asif (2014) and Persson (2009) and confirms the notion that stakeholder knowledge must have an influence on sustainable construction practice. If these skills can be enriched and better managed by company training programmes, there is scope for improving general practice and project sustainability goals.

Communication and collaboration are often factors that go hand in hand when discussing stakeholder management (Gunduz and Almuajebh 2020). The response for question S2, “Communication between stakeholders is vital for effective coordination” had a mean average of 4.51, and question S3, “Effective coordination between stakeholders makes working sustainably easier” averaged at 4.29. The obvious finding here is that most participants are aware of the importance of communication and collaboration between stakeholders. However, not all participants were certain about the effects that coordination could actually have on project sustainability. It should be considered that the wording of the question may have misled the result. Whilst 4.29 is a value that equates to general agreement, had the question been reworded to “Effective coordination between stakeholders reduces project waste and aids the meeting of more project sustainability goals”, there may have been a very different result. It could be argued that effective coordination and working more sustainably is not necessarily easier, but is simply of urgent matter, the same thoughts are provided by Chan et al (2021). Question S6 received an average of 4.36, “Stakeholders could work together to increase the sustainability of construction projects”. The opinion of the sample therefore suggests agreement that stakeholders do have the ability to take action and make more of an impact on projects, however most likely need to be coordinated more effectively before that can begin to happen (Maqbool et al. 2022b).

The findings of questions S4 and S5 suggest that contracts could potentially be used to change the way stakeholders interact with each other. There was overall agreement that transparency may encourage trust and therefore collaboration between stakeholders. If this is integrated into the contract from the very start of the engagement, it could facilitate the success of many project goals (Pishdad-Bozorgi and Beliveau 2016).

#### Project management factors impacting sustainable urbanisation

The lowest mean average response value in the questionnaire was 3.01 for question PM1, “There are enough skilled and experienced workers in construction”, which is in the Project Management related category. The results for this question had a high standard deviation value and scored generally low overall mean value, which suggests that there are not enough skilled or experienced workers in the construction industry. This could indicate that staff need more thorough training programmes or a better quality of education generally (Cebrián et al. 2020). Since over 50% of the sample was at least masters educated, it does not seem likely that lack of education in the sample is the problem. However, almost 50% had also only been working in the industry for over 5 years; therefore, the demographics suggest that the sample was relatively young or might inexperienced to modern and innovative methods of sustainability despite higher education. This indicates that more research is needed into the skills of the current workforce and their education levels comparatively, in order to assess if there is more value in higher education or learned knowledge gained through work experience.

On analysis of the Pearson correlation matrix, the most significant correlations were between Project Management factors and Energy factors, as well as Stakeholder factors and Project Management factors. The highest correlation values were for Stakeholder factors and Project Management factors with a value of 0.735 at significance level < 0.001.

Question PM7, “Projects would still be successful if they used more sustainable resources and prioritised waste management”, received a mean average response of 4.23, and 4.26 for question PM8, “There are more sustainable ways of working in construction, however, often they are ignored”. What this shows is a general agreement that more definitely can be done to work sustainably in construction however we potentially need more experienced staff or further research into more specific factors. Question PM6, “Me/ my organisation uses integrated software (such as BIM)”, scored relatively low value of 3.72 and a high standard deviation of 1.242. This suggests a large variance in the results as there is either uncertainty about this, or perhaps not many companies have invested in integrated software. Engagement and collaboration of stakeholders is said to increase via the use of such software (Olawumi and Chan 2018). It can therefore be recommended that organisations encourage and facilitate the uptake of integrative technology further.

#### Policy related factors impacting sustainable urbanisation

Question P4, “Integrated processes such as BIM aids sustainability of projects” within the Policy related factors, demonstrated that with a mean average of 4.25, the sample generally agreed that BIM and other integrative processes could increase sustainability in construction, supporting the point made previously about the uptake of such software (Kamari et al. 2021; Haruna et al. 2021).

Policy-related factors in general were answered relatively evenly. Question P8, “Governments should offer financial incentives/ subsidies to encourage sustainable practices”, accrued a mean average of 4.48 with a relatively low standard deviation of 0.770. This insinuates that there was less variance in the answers and therefore that most participants were in unanimous agreement, that government subsidies might aid the bid to work more sustainably. With that in mind, what can research do to put pressure on governments into aiding the construction sector? And what research can be done into increasing sustainable practice without the aid of government involvement? Mostly researches acknowledge on the government subsidies for the sustainable urbanisation practices (Al Mulhim et al. 2022; Hong et al. 2021).

Comparatively, the lowest value generated in the principal factor analysis table is − 0.468 highlighted in blue for question P3, “Higher education should focus more on sustainable practice”. The mean average response for this question was 4.37, with a standard deviation of 0.8, the data suggests that most participants agreed with the statement; however, the result is only of moderate significance. As previously mentioned, higher education improving its teachings on sustainability is only useful if workers in the industry are actively studying for roles (Cebrián et al. 2020). If employers can provide more graduate programmes or sustainability training courses, it might encourage sustainable practice via real-world experience as opposed to theory-based education. This also would be a more useful suggestion for industry professionals that are older or are more reluctant to study.

## Conclusions and recommendations

### Conclusions

This research study intended to identify and discuss challenges surrounding sustainability, climate change mitigation and energy security. It outlined the significance of the role of project management practices and its influence on sustainable urban development, discussing the key factors which may contribute to this influence. The study has undertaken a survey to gather quantitative data in a cross-sectional study to identify primary research on the subject matter. The findings were analysed using SPSS to generate inferential statistical tests and the results of which were discussed. The study has examined and built on existing knowledge surrounding the key factors involved in sustainability and construction practice.

In summary, the research data has demonstrated that there are several factors involved with sustainable practice and despite the overall agreement from participants, there is still a lack of implementation of such practice. What this means is that either the sample was too inexperienced to present an accurate understanding of the subject matter, or that sustainable practice is important and necessary, but is in the hands of higher powers. The sample agreed that government involvement is necessary in these actions moving forwards; however, further education on these topics may still be beneficial for construction industry professionals, especially those with a lower level of education, or alternatively, a higher level of education but with little work experience. Since the validity and reliability tests proved to be positive, we can say that the results do have some weight; however, there is much room for further research. To conclude, a holistic variety of factors have been examined in the study; however, a more specific framework for improving cross-discipline collaboration could be developed. If infrastructure is to become more efficient in rapidly urbanising cities, then investments need to be made in encouraging stakeholder trust, communication and collaboration as well as general awareness of sustainable practices. That investment should ideally come from firms, however governments could work with and financially aid organisations to coordinate and rejuvenate potentially polluted and highly-populated cities.

## Recommendations and future aspects of research

The research findings are equally important for policymakers, project firms’ top leadership, practicing engineers and NGOs and different welfare organisations. The important success factors are discussed in this research would provide a useful insight to be focused for the better sustainable urbanisation to avoid environmental risks. Moreover, the factors which does not show highest contributions towards environmental aspect of the urbanisation can be eliminated from the priority list to fully focus on the most needed and urgent steps. In addition to climate change mitigation, this research also provides useful hints for the energy security which is the key element of sustainable urbanisation. The key examples discussed in the literature review could be supported for the policymakers and practicing engineers to understand and implements useful actions to cope up global challenges to meet the sustainable urbanisations targets. So, the overall, the research contributions are multifold in its nature, which could be addressed by all the direct stakeholders of urban development.

The findings of this study indicate that more research is needed into the project management and stakeholder related factors that affect project sustainability. From the literature review, it was assumed that organisational factors would most likely have the strongest effect on project sustainability; however, it is difficult to assess from these results if that is the case. Further studies could be done into organisational cultures to see if there is any significant influence with a population sample that has more experienced participants, a larger sample size and potentially from a larger variation of countries, outside of the UK. Since this study discussed the rapid urban development of developing countries, it would be relevant to suggest a further study conducted over multiple different developing countries in order to see if their opinions differ. Since the organisational factor questions had the most variance, the chance of error was higher. However, it also means that there are conflicting opinions, and so a study for clarification would be useful, particularly one that assess variables within organisational cultures and their effects on sustainable practice. The study suggests that project stakeholders need further training on sustainable construction practices including concepts such as low-carbon electrification. Whilst they may be educated to a high standard, it seems that real-world experience in the construction industry might be more valuable to organisations.

The study itself was limited by the impact of the covid-19 pandemic. This is because any person to person interactions were suspended for health and safety purposes. The research itself relied mostly upon a literature review due to this, and all questionnaires had to be delivered electronically so as to prevent contamination of the virus through interviews and participant recruitment processes.

## Data Availability

Data generated or analysed during the study are available from the corresponding author on reasonable request.
